# Preliminary Findings on the Association of the Lipid Peroxidation Product 4-Hydroxynonenal with the Lethal Outcome of Aggressive COVID-19

**DOI:** 10.3390/antiox10091341

**Published:** 2021-08-25

**Authors:** Neven Žarković, Biserka Orehovec, Lidija Milković, Bruno Baršić, Franz Tatzber, Willibald Wonisch, Marko Tarle, Marta Kmet, Ana Mataić, Antonia Jakovčević, Tea Vuković, Danijela Talić, Georg Waeg, Ivica Lukšić, Elzbieta Skrzydlewska, Kamelija Žarković

**Affiliations:** 1 Laboratory for Oxidative Stress (LabOS), Ruđer Bošković Institute, 10000 Zagreb, Croatia; Lidija.Milkovic@irb.hr (L.M.); Tea.Vukovic@irb.hr (T.V.); Danijela.Talic@irb.hr (D.T.); 2 Clinical Hospital Dubrava, 10000 Zagreb, Croatia; biserka.orehovec@gmail.com (B.O.); barsicbruno@gmail.com (B.B.); tarlemarko1@gmail.com (M.T.); zmarta.km@gmail.com (M.K.); luksic.ivica@gmail.com (I.L.); 3 Omnignostica Ltd., 3421 Höflein an der Donau, Austria; franz@tatzber.at (F.T.); willi.wonisch@aon.at (W.W.); 4 Department of Pathology, Clinical Hospital Centre Zagreb, 10000 Zagreb, Croatia; ana.mataic@gmail.com (A.M.); antonia.jakovcevic@gmail.com (A.J.); kamelijazarkovic@gmail.com (K.Ž.); 5 Institute of Molecular Biosciences, Karl Franzens University, 8010 Graz, Austria; georg.waeg@uni-graz.at; 6 Department of Pathology, University of Zagreb School of Medicine, 10000 Zagreb, Croatia; 7 Department of Inorganic and Analytical Chemistry, Medical University of Bialystok, 15-089 Bialystok, Poland; elzbieta.skrzydlewska@umb.edu.pl

**Keywords:** COVID-19, oxidative stress, lipid peroxidation, 4-hydroxynonenal (HNE), inflammation, antioxidants, peroxides, free radicals, reactive oxygen species (ROS), blood vessels, lungs, immunohistochemistry, HNE-ELISA, SARS-CoV-2

## Abstract

Major findings of the pilot study involving 21 critically ill patients during the week after admission to the critical care unit specialized for COVID-19 are presented. Fourteen patients have recovered, while seven passed away. There were no differences between them in respect to clinical or laboratory parameters monitored. However, protein adducts of the lipid peroxidation product 4-hydroxynonenal (HNE) were higher in the plasma of the deceased patients, while total antioxidant capacity was below the detection limit for the majority of sera samples in both groups. Moreover, levels of the HNE-protein adducts were constant in the plasma of the deceased patients, while in survivors, they have shown prominent and dynamic variations, suggesting that survivors had active oxidative stress response mechanisms reacting to COVID-19 aggression, which were not efficient in patients who died. Immunohistochemistry revealed the abundant presence of HNE-protein adducts in the lungs of deceased patients indicating that HNE is associated with the lethal outcome. It seems that HNE was spreading from the blood vessels more than being a consequence of pneumonia. Due to the limitations of the relatively small number of patients involved in this study, further research on HNE and antioxidants is needed. This might allow a better understanding of COVID-19 and options for utilizing antioxidants by personalized, integrative biomedicine approach to prevent the onset of HNE-mediated vitious circle of lipid peroxidation in patients with aggressive inflammatory diseases.

## 1. Introduction

First cases of coronavirus disease 2019 (COVID-19), caused by severe acute respiratory syndrome coronavirus 2 (SARS-CoV-2) infection, were reported at the very end of the year 2019. In 2020, the virus has spread worldwide, causing pandemics as never seen before. Since the COVID-19 outbreak, scientists are trying to understand the pathology of the disease in order to improve the patient’s outcome. While only a few drugs were approved by the Food and Drug Administration as appropriate to treat patients with COVID-19, the first vaccines are implemented worldwide according to the urgent criteria, occasionally causing inexplicable side effects. Major complications of severe COVID-19 infection include acute respiratory distress syndrome (ARDS), sepsis, and multiple organ dysfunction or failure (MOF) [1]. Each of these severe disorders is associated with inflammation and excessive generation of reactive oxygen species (ROS), affecting body redox balance, thus causing oxidative damage of macromolecules under the pathophysiological process of oxidative stress (OS). Therefore, the potential use of antioxidant treatments to prevent COVID-19 progression have been already suggested [2,3,4].

One of the underlying mechanisms of organ damage in COVID-19 could be mitochondrial dysfunctions resulting in excessive production of ROS, triggered by inflammatory response to SARS-CoV-2 infection, hence resembling other types of sepsis characterized by severe inflammation [5]. There are various pathophysiological sources of OS, causing either acute or chronic disbalance of pro- and anti-oxidants, many of which do not need to result in severe health disorders unless they induce lipid peroxidation (LPO). Namely, under such aggressive OS, polyunsaturated fatty acids (PUFAs, notably ω-6 fatty acids or n-6 fatty acids, especially arachidonic and linoleic acid) in cellular biomembranes become targets for ROS-induced damage, triggering a self-catalyzed, non-enzymatic chain reaction of LPO. Thus, damaged cells in the affected tissues, together with erythrocytes in blood, generate ROS, and release of free iron, which can consequently amplify LPO through Fenton reactions and promote LPO and ferroptosis [6], resulting in membrane dysfunction/destruction and MOF in COVID-19 patients [7]. Indeed, the autopsy findings in COVID-19 patients revealed a ferroptosis signature in the epicardium and myocardium in the area of severe SARS-CoV-2 myocarditis associated with generalized lipid peroxidation [8]. Similarly, a pilot study on oxidative stress in COVID-19 patients hospitalized in an intensive care unit (ICU) for severe pneumonia has indicated systemic OS in critically ill COVID-19 patients manifested by increased LPO and deficits of antioxidants [9].

Final products of LPO are reactive aldehydes that have a longer lifetime (minutes to hours) than is the lifetime of ROS (nano to milliseconds) and might accordingly represent better biomarkers of SARS-CoV-2 induced OS. Among the LPO-derived reactive aldehydes, the most bioactive seems to be 4-hydroxynonenal (HNE). Namely, HNE acts as “second messenger of free radicals”, spreading OS and LPO even in the absence of ROS, accumulating in blood vessels in an age-dependent manner [10,11]. The affinity of HNE to bind to proteins, modifying their structure and function and acting dynamically in a concentration-dependent manner not only as a cytotoxic product of LPO, but also as a signaling, regulatory molecule, makes HNE a valuable bioactive marker for various diseases associated with OS [12,13] and their respective therapies [14], suggesting it could also be a predictive biomarker of SARS-CoV-2 induced OS. In favor of our assumption are recent autopsy findings of HNE in COVID-19 patient [8] and a positive correlation of HNE expression with the functional receptor of SARS-CoV-2, angiotensin-converting enzyme 2 (ACE2), also suggesting significant involvement of this particular LPO product in COVID-19 pathogenesis [15].

Therefore, to test our hypothesis that the onset of the vicious circle of LPO mediated by HNE might be relevant for the pathogenesis of COVID-19 and its outcome, we used genuine enzyme-linked immunosorbent assay (ELISA) specific for the HNE-protein adducts in the blood of critically ill patients who survived and those how were killed by aggressive COVID-19. The same monoclonal antibody used for the HNE-ELISA was eventually used for immunohistochemistry of lungs of some deceased COVID-19 patients.

## 2. Materials and Methods

### 2.1. Patients

This study was done upon the ethical approval 2020-1012-13 of the Clinical Hospital Dubrava in Zagreb, serving as the national center for COVID-19, thus, providing medical care for patients suffering from the most aggressive COVID-19. Only patients who signed the informed written consent and who were hospitalized at the ICU for the period long enough to collect three samples of blood starting on the first day after admission, followed by the blood sampling every second day afterward.

Of the 21 patients involved (11 men and 10 women of average age 65.3 ± 14.6 years), fourteen patients have recovered, while seven died. Although survivors were on the average ten years younger than patients who died (62.0 ± 14.7 years vs. 71.9 ± 12.4), there was no significant difference between them in respect to age (*p* > 0.1), comorbidities (3 patients had diabetes mellitus type 2, one passed away, while two survived), clinical treatment or laboratory parameters monitored.

### 2.2. Laboratory Analysis

For the analysis of biochemical and immunochemical parameters, samples of blood were centrifuged at 1370× *g* for 10 min and sera were analysed immediately after centrifugation or were stored at −80 °C to be analysed afterwards.

Leukocytes were counted in EDTA-anticoagulated blood on the DxH 800 Hematology Analyzer (Beckman Coulter, Tokyo, Japan). Concentrations of C-reactive protein (CRP) and ferritin were measured using immunoturbidimetric assays on the AU 5800 analyzer (Beckman Coulter, Tokyo, Japan). Lactate dehydrogenase, EC 1.1.1.27 (LDH) activity was measured using a kinetic UV test on AU 5800 analyzer (Beckman Coulter, Tokyo, Japan). Levels of interleukin 6 (IL-6) and Procalcitonin (PCT) were measured using chemiluminescent immunoassays on the UniCel DxI 800 Access Immunoassay System (Beckman Coulter, Tokyo, Japan).

An enzymatic assay was used to test ROS scavenging of sera using uric acid standards as described before [16]. In parallel, a complementary assay for total serum peroxides was done [16] using hydrogen peroxide standards. However, only results for serum peroxide levels were obtained, because for the majority of sera samples, total antioxidant capacity was below the detection limit equivalent to 11.7 μM of the uric acid standard. Sera samples were also used to determine the titer of the autoantibodies against oxidized low-density lipoproteins (LDL) using the oLAB ELISA as described before [17].

Parallel to the sera sampling, the EDTA plasma samples were prepared for the HNE-ELISA analysis done as described before [18,19] using a genuine monoclonal antibody specific for the HNE-histidine (HNE-His) epitopes.

### 2.3. Immunohistochemistry

Finally, immunohistochemical analysis using the same monoclonal antibody, as in the case of the HNE-ELISA, was done for the tissue specimens obtained by autopsy of patients that eventually passed away at the University Hospital for Infectious Diseases, Zagreb. Formalin-fixed, paraffin-embedded tissue specimens of lungs were analyzed by pathologists with expertise in the field, as described before [13,20]. The 3,3′-Diaminobenzidine tetrahydrochloride (DAB) was used as chromophore giving brown colored reaction in case of positive immunohistochemical reaction for the HNE-His, with blue-colored contrast staining of hematoxylin.

### 2.4. Statistics

Comparison of the mean values of the HNE-protein adducts in plasma between the two groups of patients was carried out using the *t*-test, the differences between the incidence of patients with static levels and those with dynamic changes of the HNE levels were compared between survivors and the deceased patients by chi-square test, while correlations between biomarkers analysed in comparison to the HNE levels in plasma were done by the Pearson *R* test.

## 3. Results

**Figure 1:**
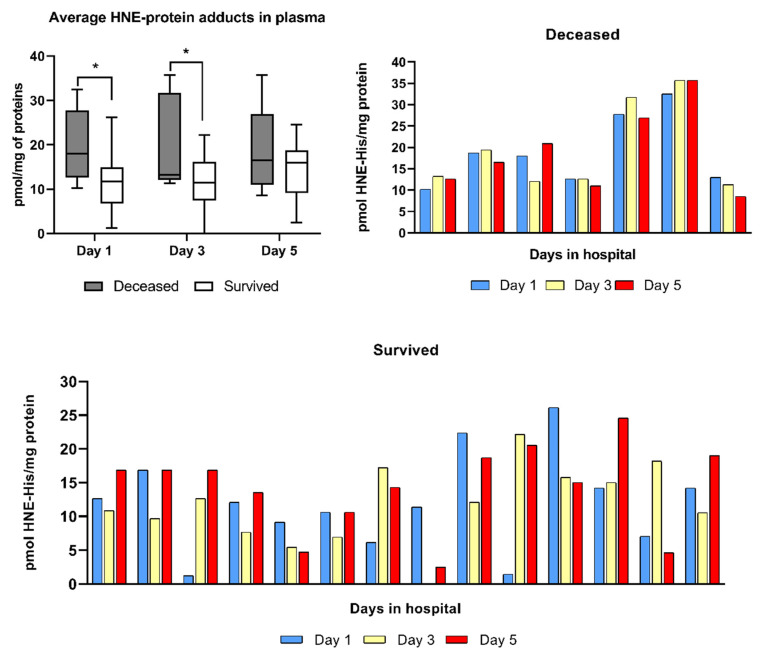
Values of the HNE-protein adducts determined in the EDTA-plasma of COVID-19 patients by the genuine HNE-ELISA specific for the HNE-His adducts. The amounts of the HNE are presented in pmol/mg protein values, while the asterisk indicates a significant difference (*p* < 0.05) between the average values determined for the plasma of survivors and for the deceased COVID-19 patients.

The analysis of HNE levels in the EDTA-plasma of patients analyzed by the genuine HNE-ELISA using a non-commercial monoclonal antibody specific for the HNE-His epitopes revealed significantly higher (*p* < 0.05) average plasma values of HNE-protein adducts in the blood of the deceased patients than in the blood of survivors for the first days (1–3) in hospital (Figure 1).

However, values of HNE-His detected in the plasma of survivors slightly increased on the fifth day, reaching the levels that were not significantly different from the values of the deceased patients indicating that the high levels of the HNE-protein adducts in plasma were not the only HNE-related parameter predicting the lethal outcome of aggressive COVID-19. Namely, the individual levels of HNE determined at different time points in the blood of patients have shown that the levels of HNE were stable in the blood of patients who died, but have shown dynamic variations in the blood of survivors revealing significantly different patterns between the two groups (chi-square *p* < 0.01). Namely, 6/7 patients who passed away did not show prominent variations of the HNE levels in the blood collected at different time points (as an exemption, we consider the 3rd patient from the left side of the respective upper graph, who had a transient decrease of HNE levels in the blood on the third day), while relatively stable, although gradually decreasing levels of HNE were observed only in 1/14 survivors (the 5th patient from the left side on the bottom graph). Hence, these findings suggest that survivors had active oxidative stress response mechanisms reacting to the COVID-19 aggression, which were not efficient in the patients who died.

The individual variations of the HNE levels in the blood were compared using Pearson correlation with all available physical and laboratory parameters of the patients, but due to the relatively small number of patients per group, the R values were significant for the deceased patients only for HNE and IL-6 (R-0.83, *p* < 0.05) on the first day after admission to the ICU. Although trends of differentially positive vs. negative correlations were observed at different time points for other parameters between survivors and the deceased patients, due to the lack of statistical significance, these trends are not presented.

However, because of possible high importance, we wish to say that the persistent negative correlation trend between the HNE levels in the blood and the levels of the autoantibodies against oxidized LDL (oLAB) was observed only in the blood of the deceased patients, while for survivors significantly positive correlation (R 0.54, *p* < 0.05) was observed on the last day of evaluation, followed by the recovery from COVID-9. Similarly, a trend of negative correlation was also observed between the HNE levels and the total peroxide levels in sera of the patients who died but were gradually lost in the terminal stage (day 5), while such a trend was not observed for survivors.

It should be mentioned here that the complementary assay for the total antioxidant capacity was also carried out, but the levels of antioxidants were too low to be determined (bellow detection limit) for the majority of samples in both groups, suggesting again that OS is one of the major pathogenic factors in aggressive COVID-19, for which patients needed additional oxygen support at ICU.

**Figure 2:**
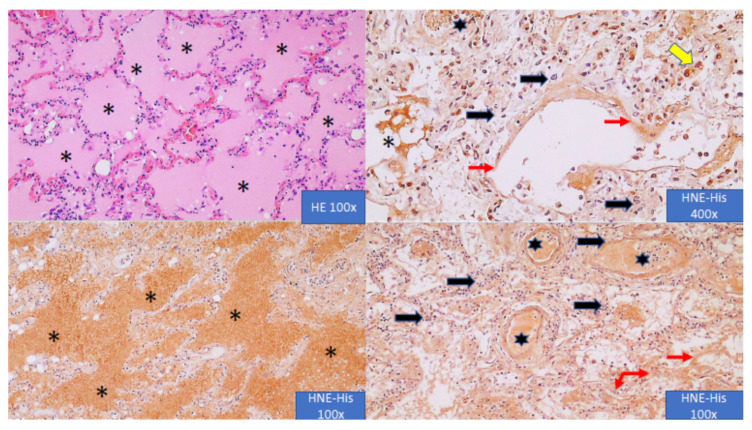
The immunohistochemical appearance of HNE in the lungs of the deceased COVID-19 patient. Eosinophilic (HE) liquid of edema in the alveoli (asterisks) shows abundant HNE content (HNE-His giving brown-colored immunopositive reaction of DAB staining). However, inflammatory mononuclear cells in the alveoli and in their septa (black arrows) are mostly negative for HNE (only blue-colored hemotoxylin contrast staining is present), with some macrophages are positive for HNE (yellow arrow). On the contrary, blood vessels (both their walls and the blood content, indicated by the black stars) are HNE-positive, as are the hyaline membranes on the surface of the alveolar septa (red arrows). Conclusion—HNE might be responsible for the fatal outcome, but more likely due to the systemic, vascular oxidative stress (sepsis-like, originating from the blood), not due to the pulmonary inflammatory cells, which are mostly negative for HNE.

Microphotographs presented in Figure 2 show the immunohistochemical appearance of HNE-His adducts in the lungs of the deceased COVID-19 patient.

Immunohistochemistry was done by the same monoclonal antibody used for the HNE-His ELISA thus detecting the same epitopes on the proteins modified by HNE due to the onset of OS in the blood and in the lungs. As can be seen, the cell-free inflammatory edema in the lungs contained abundant amounts of HNE-protein adducts. Similarly, blood vessels were loaded with HNE, as were the hyaline membranes suggesting that abundant HNE might be responsible for the vicious circle of OS in COVID-19, leading to the fatal outcome. However, pulmonary OS was probably not caused by an oxidative burst of inflammatory cells in the lungs, which were mostly negative for HNE, but was a crucial component of systemic OS spreading HNE by blood through the entire organism.

## 4. Discussion

While excessive inflammation plays an important role in aggressive COVID-19, the relevance of this generated oxidative stress is uncertain, mostly due to the short lifetime of free radicals and the complexity of the disease itself [21,22,23]. On the other hand, reactive aldehyde HNE, the particular LPO product of PUFAs, which regulates the sensitivity of cells to OS and their adaptation acting as the second messenger of free radicals and a signaling molecule, has a longer lifetime mostly because of high affinity for binding to proteins [24,25]. This pilot study revealed the abundant presence of the HNE-protein adducts in the lungs of patients who died from aggressive COVID-19. The HNE-protein adducts were also higher in the blood of these patients than in the blood of the equally-treated critically ill patients who survived COVID-19 even five days before the death/recovery and were correlated with the levels of the oxygen saturation, inflammatory parameters, and the other parameters of oxidative stress analyzed. Due to the relatively small size of the group of deceased patients (*n* = 7), the only significant correlation in the group of the deceased patients was observed between HNE and IL-6 levels upon admission to the ICU and was strongly negative (*R*-0.83). This is not surprising because HNE can act as a signaling molecule suppressing IL-6 production [26], but it is worth mentioning that it was only transient and was only observed for the patients who eventually died from COVID-19. In spite of high levels of *R*, which were mostly not significant due to the relatively small number of patients in the pilot study, obvious trends were observed showing a lot of differences between patients who survived and patients who died, which deserve further studies involving more patients.

Among other aspects of COVID-19 related OS and LPO further, studies should evaluate the immune response to oxidatively modified proteins, as we have observed a trend of negative correlation between HNE-protein adducts and the titers of autoimmune antibodies generated against oxidized LDL, only in the patients who died. Although HNE-ELISA determines mostly HNE bound to histidine residues of serum albumin, the assay also detects HNE-His present on the other sera proteins [19,27]. Since HNE-His is also a major epitope of the oxidized LDL [27], our results suggest the involvement of the immune system in the fight with toxic mediators of OS, notably HNE in COVID-19 patients. Moreover, the observed constantly negative trend of correlation between HNE-ELISA and oLAB assays in non-survivors might be interpreted as exhaustion of the immune system, which cannot produce specific anti-OS-IgG in sufficient amounts and should be further studied. On the other hand, HNE is also binding to the histidine moiety of angiotensin [27], which, together with the fact that HNE interferes with bioactivities of angiotensin [28,29] and is positively correlated with the ACE2 [15], increase the need for further research on HNE in the pathogenesis of COVID-19.

Immunohistochemistry revealed abundant HNE-protein adducts, especially in the blood vessels and inflammatory edema of the lungs affected by COVID-19. Surprisingly, inflammatory, mononuclear cells in the alveoli and in their septa were HNE-negative in spite of the fact that for inflammatory cells, HNE acts as a signaling molecule and pleiotropic regulatory factor, while macrophages, which were only exceptionally HNE-positive, are otherwise known to generate ROS and abundant HNE [30,31]. These findings suggest systemic, vascular oxidative stress in COVID-19 patients and the spread of HNE in the form of protein adducts by blood. That is in agreement with findings of toxic HNE-protein adducts accumulation in atherosclerotic blood vessels, proportional to the age, but in potentially reversible form of histidine adducts [11,32,33]. Although in our pilot study there was no significant difference between the age or patients who survived and those who died, it should be mentioned that survivors were, on average, ten years younger. Our previous research on the atherosclerotic aorta revealed that the accumulation of the HNE-protein adducts reaches its maximum around the age of 65 [11], which was the average age of our patients taken together. Hence, we can hypothesize that the age of patients might play an important role in the survival of COVID-19 also due to the age-dependent accumulation of HNE in the wall of blood vessels, while the possible release of toxic HNE from the blood vessels into other tissues should be further studied, especially because the similar accumulation of the blood-originating HNE was also revealed in abdominal adipose tissue of obese people with metabolic syndrome [34]. This assumption deserves further research, which could increase our knowledge on the oxidative stress response not only to COVID-19 aggression but also about other stress- and age-associated diseases for which HNE is known to be an important factor of pathogenesis or hormesis [35,36,37,38].

Finally, it should be mentioned that trend of negative correlation of HNE in the blood and the total serum peroxides observed in our study associated with undetectable serum antioxidant capacity (according to the TAC assay) of COVID-19 patients resembles findings of other studies and points to the high relevance of antioxidants in defense against aggressive COVID-19. Among potential antioxidants present in tissues, including blood, uric acid is often neglected, although it contributes a lot to the TAC analysis of sera, for which we even used uric acid as standard. Although we did not evaluate urate levels in the blood of our patients, further studies should do that with respect to the possible onset of ischemic/reperfusion injury that might cause systemic oxidative stress generating uric acid.

## 5. Conclusions

If further studies confirm our preliminary findings, novel options for the introduction of an integrative biomedicine approach to COVID-19 and other severe inflammatory diseases could be developed based on personalized medicine and the careful use of the most promising antioxidants that could regulate HNE metabolism and its bioactivities. It is likely that such options will include the use of lipid-soluble antioxidants to prevent the onset of LPO, such as tocopherol and lycopene, but together with water-soluble antioxidants, such as vitamin C, micronutrients needed to maintain physiological functions of enzymatic antioxidants (such as zinc and selenium) and eventually HNE-scavengers resembling GSH—the dominant physiological scavenger of HNE, such as those based on N-acetylcysteine, together with other credible natural or synthetic regulators of OS and LPO in particular.

## Data Availability

Data is contained within the article.

## Abbreviations

Coronavirus disease 2019 (COVID-19); 4-Hydroxynonenal (HNE); severe acute respiratory syndrome coronavirus 2 (SARS-CoV-2); acute respiratory distress syndrome (ARDS); multiple organ dysfunction or failure (MOF); oxidative stress (OS); reactive oxygen species (ROS); lipid peroxidation LPO); polyunsaturated fatty acids (PUFAs); low-density lipoproteins (LDL); enzyme-linked immunosorbent assay (ELISA); angiotensin-converting enzyme 2 (ACE2); HNE-histidine (HNE-His); intensive care unit (ICU); interleukin 6 (IL-6); immunoglobulin G (IgG), hematoxylin/eosin staining (HE); 3,3′-Diaminobenzidine staining (DAB).
